# A comparative case control study of the determinants of clinical malaria in The Gambia

**DOI:** 10.1186/1475-2875-13-306

**Published:** 2014-08-08

**Authors:** Joseph Okebe, Julia Mwesigwa, Eugene L Kama, Serign J Ceesay, Fanta Njie, Simon Correa, Kalifa Bojang

**Affiliations:** Medical Research Council Unit, Fajara, The Gambia; National Institute of Public Health, Conakry, Guinea

**Keywords:** Malaria, Risk, Socio-economic

## Abstract

**Background:**

The massive deployment of life saving malaria interventions has not only resulted in a decline in disease burden but a change in the risk of infection and disease. The study reassesses the importance of known risk factors and reviews demographic and socio-economic determinants of malaria risk in the population.

**Methods:**

This was a case-control study involving 150 children with test-confirmed malaria infection recruited from the outpatient clinics of three health facilities (cases) in the Greater Banjul area, The Gambia. One hundred and fifty controls, negative for malaria were matched on age, residence. Information was collected from respondents on the use of long lasting insecticidal nets, occupation, housing structure, knowledge of malaria and socio-demographic factors.

**Results:**

The mean age of study participants was 6.8 (SD 3.3) years with 147 (49%) being males. Significant determinants of malaria risk were parent’s occupation: mother as trader (OR 0.18, 95% CI 0.04 - 0.73, p = 0.017), father as trader (OR 0.02, 95% CI 0.002- 0.193, p = 0.001), civil servants (OR 0.04, 95% CI 0.008- 0.257, p =0.001) or handyman (OR 0.03, 95% CI 0.005- 0.182, p < 0.001). Children sleeping in rooms with windowpanes had a 76% reduction in their odds of malaria (OR 0.24, 95%CI 0.07- 0.82, p = 0.022.

**Conclusion:**

Household socio-economic status plays an important role in management of illnesses. The ability of mothers to engage in an occupation increases household resources to access healthcare and on time. The balance between the type of mother’s occupation and her time available to supervise the child is an interesting emerging issue that needs further investigation.

## Background

The scaling up of effective interventions, such as long-lasting insecticidal nets (LLINs), indoor residual spraying (IRS), treatment decisions based on microscopy or rapid diagnostic tests (RDTs) and, artemisinin-based combination therapy (ACT) has led to a substantial decline in global malaria burden [1]. Nevertheless, malaria remains the most common cause of outpatient consultations in sub Saharan Africa. In addition to the direct effect on mortality and morbidity, malaria has serious negative macroeconomic effects on the continent [2]. Malaria is distributed disproportionately with reported determinants of transmission, including; area of residence [3], overcrowding [4], maternal education [5], knowledge about prevention of malaria [6, 7] and socioeconomic factors [6]. Studies indicate that there is less malaria in urban compared to rural areas [8, 9] and this is attributed to improved housing and drainage [10] which reduce mosquito breeding sites and better access to health facilities for prompt treatment of disease [11].

A previous study carried out in The Gambia showed that poor quality housing, overcrowding, travel to rural areas and household economic index, but not education level of parents or guardians were associated with the risk of malaria [6]. Since then, the country has seen improvements in living standards and health infrastructure: scaling up of LLINs distribution, a change of first-line malaria treatment to ACT and strong educational campaigns aimed at promoting behavioural change. This has culminated in a decline in many malaria indices [12]. As the epidemiology of the disease evolves towards increasing heterogeneity, the relative importance of individual risk factors are likely to change and targeted interventions will probably be more efficient. This case control study was conducted in an urban setting to understand the effect of these changes and re-evaluate identified risk factors for clinical disease during the malaria transmission season in Gambian children.

## Methods

### Study design

This was a matched case–control study to explore associations between participant/household risk factors and uncomplicated malaria from October to December 2012. The study was conducted in three health facilities in the Western Region of The Gambia; Sukuta Health Centre, Sukuta; Medical Research Council Clinic, Fajara and Edward Francis Small Teaching Hospital, Banjul. The climate in The Gambia is typically sub-Sahelian with a short wet season from May/June to October/November and a long dry season for the rest of the year. Mean monthly temperatures range from 25°C to 29°C with the relative humidity varying from 40% in the dry season to over 80% in the rainy season. About half of the population live in the western coastal area of the country and the economy of the area depends on trade and tourism with subsistence farming mainly during the rainy season. Malaria transmission is seasonal starting shortly after the onset of the rains [13]. The country has an established primary health care system with a number of private and government clinics available to the communities though most attend government clinics because services are cheaper. Since 2011, guidelines on malaria case management in the country recommend treatment based on parasitological diagnosis with microscopy or RDTs. Artemether-lumefantrine (AL) is the first-line anti-malarial in the country and treatment is provided free-of-charge in the public sector.

Cases were children aged six months to 12 years with confirmed malaria attending the outpatient clinics of the study clinics. To select a route along which the search for a suitable control will follow, a trained field staff stood at the doorstep of the index case’s house and spun a pen on the ground. The team then visits households in the direction of the long axis of the pen visiting until a potential age-matched child is found. On finding such a child, additional criteria for recruitment; residence in the area in the previous six months, no history of malaria treatment in the preceding two weeks and a negative rapid antigen test for malaria are checked after obtaining a written consent from the child’s mother or other responsible adult available. On confirming eligibility, information on risk factors of interest was collected using a pre-tested questionnaire. These included information on parents’ educational status, ownership and status of bed nets, number of sleeping rooms and individuals sleeping in each room and, housing characteristics; type of roof/wall/windows. Household socioeconomic status was assessed by the occupation of parents and source of funds available for malaria treatment.

### Data handling and analysis

Questionnaires were manually checked for completeness and the data double entered into an SQL database using Microsoft Office Access front-end software. A univariate analysis using conditional logistic regression was performed to identify independent variables that were strongly associated with the risk malaria. These were included in a stepwise multivariate regression model to identify those that remained significant after adjusting for other variables. All the analyses were done using Stata 12 (Stata Corp, Tx) and the level of significance was set at p <0.05.

### Ethical review

The study was reviewed and approved by the Gambia Government/MRC joint Ethics Committee (SCC 1263).

## Results

One hundred and fifty pairs of children, matched on age and residence, were recruited between September and December 2012. The table shows results of the univariate and multivariate analysis of demographic, economic and household risk factors for malaria (Table 1). The mean age of participants was 6.8 (SD 3.3) years; cases 6.8 (SD 3.3), controls 6.7 (SD 3.4) with 147 (49%) being males and the proportion of males were similar in the case and control groups. One hundred and five (70.9%) cases and 102 (68.0%) controls were more than five years of age.

**Table 1:** **Demographic, socioeconomic and household variables associated with clinical malaria in The Gambia**

Variable*		Case *n*(%)	Control *n*(%)	Crude OR (95% CI)	P	Adjusted OR (95% CI, n = 294)	P
Age group (n = 298)	≤ 5 years	43 (29.1)	48 (32.0)	1		1	
	> 5 years	105 (70.9)	102 (68.0)	1.67 (0.40-6.97)	0.484	0.74 (0.06-8.51)	0.811
Level of mother’s education (n = 298)	No formal	87 (58.4)	87 (58.4)	1			
	Quran	16 (10.7)	9 (6.0)	1.87 (0.68-5.15)	0.22		
	Primary	12 (8.1)	15 (10.1)	0.84 (0.38-1.83)	0.66		
	Post primary	34 (22.8)	38 (25.5)	0.89 (0.49-1.62)	0.71		
Mother’s occupation	House wife	83 (55.3)	91 (60.7)	1		1	
	Farmer	23 (15.3)	7 (4.7)	5.47 (1.72-17.36)	0.004	2.91 (0.57-14.77)	0.281
	Trader	6 (4.0)	40 (26.7)	0.22 (0.09-0.54)	0.001	0.18 (0.04-0.73)	0.017
	Civil Servant	7 (4.7)	3(2.0)	2.57 (0.62-10.76)	0.195	6.74 (0.08-43.83)	0.394
	Others	31 (20.7)	9 (6.0)	5.58 (1.79-17.36)	0.003	5.95 (0.61-57.77)	0.124
Father’s occupation	Seasonal worker	89 (59.3)	8 (5.3)	1			
	Farmer	15 (10.0)	11 (7.3)	0.40 (0.13-1.21)	0.104	0.19 (0.04-0.91)	0.038
	Trader	3 (2.0)	29 (19.3)	0.01 (0.001--0.06)	<0.001	0.02 (0.002-0.193)	0.001
	Civil Servant	10 (6.7)	31 (20.7)	0.02 (0.01-0.10)	<0.001	0.04 (0.008-0.257)	0.001
	Handyman	33 (22.0)	71 (47.4)	0.04 (0.01-0.13)	<0.001	0.03 (0.005-0.182)	<0.001
Source of funds for illness	Father	100 (66.67)	61 (40.67)	1		1	
	Mother	15 (10.0)	21 (14.0)	0.45 (0.21-0.99)	0.05	0.29 (0.06-1.37)	0.118
	Others	19 (12.67)	26 (17.33)	0.33 (0.15-0.73)	0.01	0.24 (0.05-1.41)	0.072
	Both parents	16 (10.67)	42 (28.0)	0.18 (0.09-0.39)	<0.001	0.05 (0.008-0.319)	0.001
Type of floor	earth	7 (4.67)	12 (8)	1			
	tile	44 (29.33)	39 (26.0)	1.96 (0.68-5.59)	0.21		
	cement	99 (66)	99 (66)	1.75 (0.63-4.85)	0.28		
Type of window (n = 299)	Boarded windows	70 (46.67)	27 (18.12)	1			
	windows with panes	80 (53.33)	122 (81.88)	0.27 (0.16-0.47)	<0.001	0.24 (0.07-0.82)	0.022
Number of bed rooms	single	19 (12.7)	14 (9.3)	1			
	2-4 rooms	108 (72.0)	126 (84.0)	0.69 (0.32-1.48)	0.339		
	over 4	23 (15.3)	10 (6.7)	1.80 (0.59- 5.53)	0.301		
Wall type	Mud	53 (35.3)	46 (30.7)	1			
	Cement	97 (64.7)	104 (69.3)	0.78 (0.46-1.32)	0.355		
LLIN in sleeping room (n = 266)	No	33 (24.44)	17 (12.98)	1			
	Yes	102 (75.56)	114 (87.02)	0.39 (0.18-0.85)	0.02		
Slept under LLIN last night (n = 269)	No	45 (34.09)	33 (24.09)	1			
	Yes	87 (65.91)	104 (75.91)	0.55 (0.29-1.01)	0.06		

*Unless specified, n = 300 observations.

Among the cases, 22.8% of mothers had formal education beyond the primary level. In the univariate analysis, children whose mothers were farmers (OR 5.46, 95% CI 1.72, 17.36, p = 0.004) had an increased odds of malaria compared to children of housewives. In contrast, in the multivariate analysis, children of mothers involved in a trade (OR 0.18, 95% CI 0.04-0.73, p = 0.017) and fathers with a reported occupation; traders (OR 0.02, 95% CI 0.002- 0.193, p = 0.001), civil servants (OR 0.04, 95% CI 0.008- 0.257, p =0.001) and handymen, (OR 0.03, 95% CI 0.005 - 0.182, p < 0.001) had reduced risk of malaria. In addition, the odds of disease were lowest where funds for healthcare were provided by both parents (OR 0.05, 95% CI 0.008- 0.319, p = 0.001).

A principal component analysis of the collected house characteristics; wall type, type of window, type of floor was used to generate a descriptive of the quality of the housing in the area. Based on this, the type of wall was the best indicator of the quality of the house followed by the type of window although, a strong evidence of an association was seen only in the type of window in the houses of cases and controls (Chi^2^ 27.79, P <0.001). Bed net coverage was high among both case (75.6%) and control groups (87.0%) and among those with LLINs, 87 (65.9%) cases and 104 (75.9%) controls reportedly slept under one the previous night. LLIN ownership (OR 0.39, 95% CI 0.18- 0.85, p = 0.02) and a history of use the previous night (OR 0.55, 95% CI 0.30, 1.02, p = 0.06) were associated with a reduced odds of malaria. Of interest was that children who slept in rooms with windowpanes had a lower risk of malaria (OR 0.24, 95%CI 0.07, 0.82, p = 0.022). The floor type and number of sleeping rooms were not associated with malaria.

## Discussion

The study investigates the socio-economic and demographic factors that characterise malaria risk in a setting with a significant decline in case burden. A number of changes in the demographic characteristics of the study population suggest an improvement in the socio-economic status of the population since the previous study. At least 42% of women had some formal education (including those with Quaranic education) compared to 10% in the previous study carried out in The Gambia [6]. Malaria-based communication tools are designed for a broad audience and incorporate the spectrum of educational and social strata in the community. Estimation of education level did not effectively discriminate risk of disease in the study as malaria awareness: causes and prevention were equally high in both groups and was not different between the groups. This finding was similar to those of two previous studies in The Gambia [6] and Yemen [14] although attainment of education standard of at least primary school level conferred significant protection from the risk of developing clinical malaria in a case control study in Peru [15].

Household socio-economic status plays an important role in management of illnesses because individuals with higher income have more resources to access healthcare on time. In this study, there was an interesting association between the occupation of the mother and the odds of disease with the odds being higher in farmers and civil servants but low in traders. One possible explanation would be the relationship between the time of contact between the child and mother and the resources available at her disposal to source for care. Farmers and civil servants are likely to spend long periods at work and therefore have less time available with their children. Similarly, Guthmann and colleagues observed that children of agricultural workers were less supervised because caregivers spend most of their time away from home [15].

Although traders and civil servants may have similar resources available to them, traders have better access to these resources and more control over their time. Children whose fathers had long-term jobs had reduced odds of malaria and this in line with role of men in Gambian societies where the fathers normally provide the funds to access health care. In contrast, a study in Yemen showed that the occupation of parents did not affect the risk of malaria. In that study however, indicators of socio-economic status were not sufficiently discriminatory in the population [14].

In The Gambia, the main mosquito vector shows preference for indoor feeding, therefore, type and number of entrances to the house could influence the risk of being bitten by mosquitoes and subsequently infected with malaria. Thus, the type of windows and the availability of preventive measures such as mosquito screens on doors and windows would be important determinants of the risk of malaria in the house [16]. The rainy season is typically hot and humid so households have to balance the need for ventilation on the one hand and prevention of nuisance associated with mosquito bites on the other hand. This is however unlikely to fully explain why children who slept in rooms with windowpanes had a lower risk of malaria because of the high rates of bed net ownership in both groups. Alternatively, availability of windowpanes may reflect affordability and thus, the economic status of the household. Other studies have reported different effects of the open window and malaria risk and their findings range [14] from no effect to a significantly higher risk of malaria [7]. Children who lived in houses with brick or plastered walls had a two to six times increased odds of disease. It is likely that the brick walls would have small opening that allow mosquitoes into the house at night. Coleman and colleagues observed that that people living in traditional mud wall houses had an increased risk of malaria than those in western – style brick walls dwellings [7].

Of particular note is the risk of malaria was significantly reduced when the source of funding for health care was from both parents when compared to either of them and reflects the positive effect of the improving economic status of families especially women in the country. A previous study in the Gambia also identified the degree of financial autonomy of the caregiver, her source of revenue for health-care expenses as well as her ability to access resources as important risk factors for illness and death [17].

Bed net ownership was associated with reduced odds of disease in the univariate analysis but was not significant in the multivariate model. This is probably due to the increased bed net usage among cases and controls and its protective effect on the risk of malaria could have been cancelled out in the final model. However, Koram and colleagues [6] found that the use of bed nets was not associated with protection among children in this urban setting. However several cross-sectional studies in Tanzania have shown bed net coverage levels were independently associated with reduced risk of *Plasmodium falciparum* in households with low and high coverage, respectively, compared to those without bed nets [18, 19].

## Conclusion

The occupation of the father as well as the availability of funds in the family for health care were significantly associated with malaria in urban areas of The Gambia. Improvements in the economic status of women could contribute significantly to reducing the risk of malaria.
